# P2Y Purinergic Receptors, Endothelial Dysfunction, and Cardiovascular Diseases

**DOI:** 10.3390/ijms21186855

**Published:** 2020-09-18

**Authors:** Derek Strassheim, Alexander Verin, Robert Batori, Hala Nijmeh, Nana Burns, Anita Kovacs-Kasa, Nagavedi S. Umapathy, Janavi Kotamarthi, Yash S. Gokhale, Vijaya Karoor, Kurt R. Stenmark, Evgenia Gerasimovskaya

**Affiliations:** 1 The Department of Medicine Cardiovascular and Pulmonary Research Laboratory, University of Colorado Denver, Aurora, CO 80045, USA; derek.strassheim@cuanschutz.edu (D.S.); nana.burns@cuanschutz.edu (N.B.); vijaya.karoor@cuanschutz.edu (V.K.); kurt.stenmark@cuanschutz.edu (K.R.S.); 2 Vascular Biology Center, Augusta University, Augusta, GA 30912, USA; AVERIN@augusta.edu (A.V.); RBATORI@augusta.edu (R.B.); AKOVACSKASA@augusta.edu (A.K.-K.); 3 The Department of Pediatrics, Division of Critical Care Medicine, University of Colorado Denver, Aurora, CO 80045, USA; hala.nijmeh@cuanschutz.edu; 4 Center for Blood Disorders, Augusta University, Augusta, GA 30912, USA; USIDDARAMAPPA@augusta.edu; 5 The Department of BioMedical Engineering, University of Wisconsin, Madison, WI 53706, USA; kotamarthi@wisc.edu (J.K.); ygokhale@wisc.edu (Y.S.G.)

**Keywords:** purinergic P2Y receptors, cardiovascular diseases, endothelial cells, vasa vasorum, angiogenesis, vascular permeability, vasoconstriction/vasodilation, vascular injury, endothelial dysfunction, intracellular signaling

## Abstract

Purinergic G-protein-coupled receptors are ancient and the most abundant group of G-protein-coupled receptors (GPCRs). The wide distribution of purinergic receptors in the cardiovascular system, together with the expression of multiple receptor subtypes in endothelial cells (ECs) and other vascular cells demonstrates the physiological importance of the purinergic signaling system in the regulation of the cardiovascular system. This review discusses the contribution of purinergic P2Y receptors to endothelial dysfunction (ED) in numerous cardiovascular diseases (CVDs). Endothelial dysfunction can be defined as a shift from a “calm” or non-activated state, characterized by low permeability, anti-thrombotic, and anti-inflammatory properties, to a “activated” state, characterized by vasoconstriction and increased permeability, pro-thrombotic, and pro-inflammatory properties. This state of ED is observed in many diseases, including atherosclerosis, diabetes, hypertension, metabolic syndrome, sepsis, and pulmonary hypertension. Herein, we review the recent advances in P2Y receptor physiology and emphasize some of their unique signaling features in pulmonary endothelial cells.

## 1. Extracellular Nucleotides and Purinergic Receptors

Extracellular nucleotides (ATP, ADP, UTP) are emerging as important autocrine/paracrine regulators of the vascular, immune, and hematopoietic systems [1,2,3,4,5,6,7,8,9,10]. Under pathological conditions, such as inflammation, hypoxia, and vascular injury, high amounts of ATP can be released locally and promote vascular responses through the activation of purinergic receptors [8,9,11,12,13,14,15]. There are 19 different cell surface purinergic receptors [16], divided into P1 types (binding adenosine) and P2 types (binding ATP, ADP, UTP, β-NAD) [3,17,18]. P2 types are further divided into ligand-gated ion channels (P2X) and membrane-bound G-protein-coupled receptors (P2Y). In endothelial cells (ECs), extracellular nucleotides act through P2Y (metabotropic) receptors to regulate endothelial barrier function in both physiological and pathological conditions [1,2,3,4,5,6,19,20]. So far, eight mammalian P2Y receptor subtypes (P2Y1, P2Y2, P2Y4, P2Y6, P2Y11, P2Y12, P2Y13, and P2Y14) have been identified. Interestingly, all P2Y receptors have been shown to belong to the gamma-subgroups of class A GPCRs. P2Y1, 2, 6, and 11 are coupled to Gq. P2Y11 is also coupled to Gs, which increases the activity of adenylate-cyclase and thus increases the level of cAMP [21]. P2Y12, 13, and 14 are coupled to Gi, which inhibits adenylate cyclase and activates additional effector systems, including phosphatidyinositol-3 kinase (P3IK). P2Y4 is coupled to Gq and Gi [3,10,17,22,23]. We have recently demonstrated the expression of all P2Y receptors in lung microvascular EC (LMVEC), with P2Y6 being the most abundant and P2Y4 being the least abundant [24]. However, within the P2Y12,13,14 group, only P2Y13 has been observed in vasa vasorum EC (VVEC) [25], indicating that P2Y receptor expression may differ across EC subtypes.

The wide distribution of purinergic receptors in the vascular system, together with the expression of multiple receptor subtypes in endothelial cells, point to their physiological and pathophysiological importance. It is also becoming evident that alterations in the purinergic signaling may result in the development of a variety of neurogenerative, immune, and vascular pathologies [9,10,18]. Some additional reports indicate that extracellular ATP has been implicated in the hyperplasia and hypertrophy of arterial walls in spontaneously hypertensive rats [3], in the regulation of vascular barrier function, and the control of proliferation and migration of vascular smooth muscle cells and hematopoietic stem cells [26,27,28,29,30,31]. Purinergic antithrombotic drugs have been shown to reduce the risk of recurrent strokes and heart attacks [9]. While numerous studies demonstrated that P2 receptors are involved in the progression of lung diseases, they primarily focused on the role of ATP-induced P2X receptor activation [32,33]. The information on the role of P2Y receptors is more limited. Early studies demonstrated that ATP-induced activation of P2Y receptors is involved in acute lung inflammation and ventilator-induced lung injury [34]. More recently, we have shown that P2Y receptor agonists, ATPγS, and β-NAD enhance the lung endothelial barrier function in vitro and in vivo via activation of specific P2Y receptors [24,35,36,37].

## 2. P2Y Receptor Signaling and Endothelial Dysfunction

The vascular endothelium is a semi-selective diffusion barrier between the plasma and interstitial fluid and is critical for normal vessel wall homeostasis. In addition, due to its secretory and adhesive properties, the endothelium is recognized as an active part of the vasculature [38,39]. In terms of EC physiology, P2Y purinergic receptors are involved in the regulation of vascular tone and barrier function, activation of vascular inflammation and thrombosis, and regulation of vascular growth-all processes are tightly linked with vascular diseases. Importantly, P2Y-mediated signaling is integrated in a crosstalk with many more regulatory systems, including cytokines, growth factors, and toll-like receptor (TLR) input, and is counterbalanced by PGI_2,_ extracellular adenosine and others. As an example of stepwise activation, P2Y2 activity is most effective at stimulating EC proliferation when working in conjunction with other growth factors or cytokines [40]. Other evidence supporting this observation comes from the cancer therapy field, showing that activation of EC P2Y receptors is protective against the DNA damaging activity of cancer chemotherapies toward the cardiovascular system [41]. There is growing evidence for a new role or P2Y subtype-specific signaling in ECs in various pathophysiological conditions.

### 2.1. P2Y Receptors and Vascular Tone Regulation

#### 2.1.1. Hypertension

Vasodilation occurs via multiple mechanisms and is protective against high blood pressure, a major pathological factor in cardiovascular disease (CVD) [42]. Fluid shear stress, as found in systemic hypertension or in pulmonary arterial hypertension (PAH), exerts a negative feedback response to decrease pressure by causing local vasodilation. The mechanism involves the activation of the endothelial mechanosensitive cation channel PIEZO1, leading to ATP release and the stimulation of P2Y1,2,4,6/Gαq-PLC-Ca^2+^ pathways in the endothelium, in turn leading to eNOS-NO-mediated vasodilation [14]. Mice with endothelium-specific P2Y2 or Gαq/Gα11 deficiency lacked flow-induced vasodilation and developed hypertension accompanied by reduced eNOS activation [42]. Endothelium-specific PIEZO1 null mice do not generate high-flow-induced NO and vasodilation and, therefore, develop hypertension [14]. The small molecule activator of mechanosensitive PIEZO1, Yoda1 replicated to some degree, the effect of fluid shear stress on ECs and induced vasorelaxation in a PIEZO1-dependent manner. Activation of EC eNOS is considered paramount in EC-dependent vasodilation and is partially Ca^2+^-dependent [43]. Activation of TRPV4 channels creates localized Ca^2+^ increased-sparks in the vicinity of TRPV4-GPCR, leading to localized eNOS activation through the calmodulin-binding domain [43,44].

Mechanisms contributing to decreases in blood pressure are mediated by the activation of P2Y2. [45]. Intermediate-conductance (KCa3.1, expressed in endothelial cells) and big-conductance potassium channels (KCa1.1, expressed in smooth muscle cells), as well as components of the myoendothelial gap junction, connexins 37 and 40 (Cx37, Cx40), are all hypothesized to be part of the endothelium-dependent hyperpolarization (EDH) response. Studies in wild-type mice and mice lacking KCa3.1, KCa1.1, Cx37, or Cx40 demonstrated that loss of endothelial KCa3.1 channel and Cx40 decreased the vasodilator response to P2Y2 activation. These data indicate the presence of eNOS-NO-independent vasodilation response, in addition to eNOS-NO-dependent response [45]. In pressure-overload-induced CVD, such as that occurring in PAH and systemic hypertension, attenuation of P2Y1,2,4,6-mediated vasorelaxation has been observed, also indicating a possible connection to the increased level of the vasoconstrictor TXA_2_ [46].

#### 2.1.2. Vascular Tone and Aging

Experimental evidence points to the involvement of P2YRs in aging-dependent hypertension [47]. Aging has been found to selectively diminish vasodilation that was mediated by P2Y2, but not muscarinic and nicotinic receptors [48]. Experimental aged rats showed impaired cardiac contractility and aortic blood flow, increased vascular inflammation, and persistently activated CaMKII [49]. Endothelial cells isolated from young and aged aorta exhibit differences in cell phenotype and physiology. A giant cell phenotype is typical in senescent cells, which are far more common in aged vessels and have a reduced capacity for proliferation or vasodilation in response to vascular stress. CaMKIIδ expression is significantly increased and activated in the endothelium of aged aorta [49]. As such, CaMKIIδ could serve as an essential marker of and maybe an appropriate candidate for investigating targeted therapy at the endothelial dysfunction that accompanies the aging process [49]. Reduction of NO-mediated vasodilation in aged blood vessels was due to decreased phosphorylation of eNOS on critical Ser1177 residue, and increased ceramide-activated phosphatase 2A might be responsible for decreased eNOS Ser1177 phosphorylation [50]. Another factor in the increased occurrence of hypertension with age is P2Y6 dimerization with the angiotensin II type 1 receptor (AT1R) [51]. With age, P2Y6 expression in VSMC increases, and heterodimers with AT1R are reported. In P2Y6 null mice, chronic over-administration of AngII resulted in less cardiac hypertrophy, vascular remodeling, and lower blood pressure [51].

#### 2.1.3. Vascular Tone and Diabetes

Type 2 diabetes (T2D) is known to impair vascular perfusion through poorly defined mechanisms. It was found that diabetics have impaired vasodilator responses to purinergic, but not to muscarinic or nicotinic receptor-induced vasodilation, similar to the situation in aging [52]. In the streptozotocin-induced Type 1 diabetes (T1D) model, impaired P2Y1-mediated vasodilation has been attributed to decreased activation of eNOS-NO-PKG [53]. At the same time, P2Y1 receptor expression remains unchanged despite the frequent occurrence of receptor expression changes in diabetic disease states [54]. In general, alteration of purinergic receptor sensitivity rather than the changes in receptor expression accounts for vascular dysfunction in diabetes [55]. Additional data from the mice model demonstrated that diet-induced T2D resulted in vascular inflammation and insulin resistance, accompanied by decreased eNOS-NO activity [53,56]. Sepsis-promoted endothelial dysfunction, monitored by the ex vivo examination, was also associated with reduced activity of eNOS-NO generation [57]. Therefore, it is suggested that amplifying the eNOS-NO signaling axis might decrease the inflammation-induced vascular damage observed in several CVDs.

### 2.2. P2Y Receptors and Regulation of Oxidative Stress and Vascular Inflammation

#### 2.2.1. Oxidative Stress

Oxidative stress is a feature of many CVDs and often closely correlates with inflammation, as both professional phagocytes bound to ECs and ECs activated by cytokines/TLRs produce ROS [58]. P2Y1 stimulation produces ROS via the NADPH oxidase catalytic component, NOX2, which produce superoxide [59,60]. Oxidative stress is reported in the ED observed in sepsis [57]. P2Y1,2,4,6 probably all activate ROS production in ECs, as all activate PKC, which seems to be at least one of the critical steps to activate the superoxide generating complex of NOX enzymes [59]. Indeed, angiotensin II and thrombin, which both have virtually identical signaling mechanisms to the P2Y1,2,4,6 group, also activate ROS production in ECs via activation of PKC [61]. One can then appreciate the clinical importance of studies proclaiming that ROS are intrinsic mediators of purinergic signaling [62]. Considering this, the role of ROS (including hydrogen peroxide) in the signaling of purinergic receptors should be examined, as was shown for P2Y1 [62].

#### 2.2.2. Vascular Inflammation

Inflammation is a characteristic feature of CVD and is mediated by released cytokines, chemokines, eicosanoids, and ATP by vascular and blood cells. ECs represent a primary cellular target for many pro-inflammatory stimuli. Potent pro-inflammatory mediators, such as LPS and TNFα, significantly induce the P2Y6 receptor expression in EC in vitro and in vivo [63]. In vascular ischemic injury models, the ischemic conditions led to the activation of P2Y2 via released ATP [64]. P2Y2 was determined to have a pathogenic role by increasing local vascular inflammation and up-regulating TNFα, IL-1, and LT-α [64]. Additionally, these inflammatory conditions promoted VSMC proliferation, resulting in neo-intimal thickening, hyperplasia of VSMCs, and monocyte adhesion to EC. P2Y2 activity amplifies the release of IL-1 by a mechanism involving NFκB and Panx1 channels [65]. While overall P2Y2 effects are pro-inflammatory, it can initiate a negative feedback mechanism, upon which it down-regulates the ICAM-1 to limit inflammation in a miR-22 dependent manner [66]. P2Y4 also exhibits a pathogenic role in ischemic myocardial injury [67]. In the mouse ischemic heart model, loss of P2Y4 nucleotide receptor protected against myocardial infarction through endothelin-1 downregulation and MMP-1,-8,-9 expressions [67]. In ischemic injury, hypoxia due to poor perfusion is a significant factor of endothelial dysfunction. P2Y2 (and probably P2Y1,4,6) works together with the P1 receptor subtype A2B adenosine receptors to protect the ECs from hypoxia-induced apoptosis. This makes some sense, as A2B receptors are anti-inflammatory, and the reported effects involve the activation of the P2Y2-Gq-PI3K-Akt and the PI3K-Akt arms, well known for their cell survival action [68,69].

The P2Y11 receptor, activated by ATP, has anti-inflammatory actions, which could be implicated in EC protection in CVDs [70]. In ECs, antagonism of P2Y11 with a specific antagonist (NF157) increases the effectiveness of oxidized-LDL to promote inflammation of endothelial cells [71]. P2Y11 expression is up-regulated by oxidized-LDL activation of ECs, reminiscent of a classic negative feedback loop. NF157 decreases IL-6 and TNFα cytokine production and the activation of p38 MAPK in ECs and the consequent binding of monocytes to EC due to up-regulation of E-selectin and VCAM-1 [71]. Other reports show the anti-inflammatory effect of P2Y11 in EC, where the receptor can block the signaling activated by IL-1, TNFα, and other cytokines, particularly the activation of JNK [72]. Thus, agents activating P2Y11 could have protective therapeutic value to combat the atherosclerosis and inflammation that drives the disease.

The mechanism by which P2Y11 exerts anti-inflammatory action are not known, but P2Y11 is a dual-specificity GPCR, activating both Gq and Gs. The activation of Gs has been reported to be potentially more circumstance- or cell-type-dependent [73]. However, a significant clue is the activation of the endothelial Gs-AC-cAMP-PKA-EPAC axis, one of the most potent anti-inflammatory and barrier-protective cellular pathways. This pathway has been known for decades, but many of the key targets of (protein kinase A) PKA, those by which PKA exerts its anti-inflammatory effect, are still unclear. One target is the pro-inflammatory cytokine receptor for TNFα, which is inactivated by PKA-mediated phosphorylation leading to anti-inflammatory actions. One example is the PKA phosphorylation of TNFαR at Thr411 and, which blocks all downstream signaling [74], similar to the action of other cytokine receptors. However, this long-known phenomenon is incompletely characterized at the molecular level and deserves further investigation.

P2Y11 receptor has demonstrated anti-inflammatory effects on ECs in other settings, such as in tumors, where ECs often migrate from the tumor locus. In this setting, cytokine- growth factor-, and chemokine-driven migration can be inhibited by activation of P2Y11. Such effects require adenylate cyclase 10 isoforms of the enzyme [75,76]. In addition, activation of EPAC1, the cAMP-activated guanosine nucleotide exchange factor (GEF) for a small GTPase, Rap1, often opposes the pro-inflammatory action of RhoA-ROCK via Tiam-1 and Vav-mediated Rac activation that strengthens endothelial adherens junctions leading to the prevention of vascular leak and inflammation [77].

#### 2.2.3. Atherosclerosis

In atherosclerosis, the P2Y2 receptor apparently has pro-inflammatory actions, as ApoE-/-mice with additional deletion of P2Y2 have lower inflammatory indices in an atherosclerotic lesion, which includes lower VCAM-1 and LT-α, a member of TNFα family known to be pathogenic in the disease [78]. As such, the investigations suggest blocking P2Y2 might be therapeutic for atherosclerosis by reducing the difficulty of treating inflammation. Oxidized-LDL promotes activation of ECs by releasing ATP, auto-activating the P2Y2 receptors, which serve to facilitate leukocyte binding to the ECs, increasing inflammation in the atherosclerotic lesion [79]. This oxidized-LDL-driven inflammation appeared to involve activation of the inflammasome, and mitochondrial ROS production to generate IL-1. Additional indications of TLR9 activity were evident, probably acting as a DAMP (damage-associated molecular pattern) response [80]. Other evidence supports the pro-inflammatory phenotype of P2Y12 in atherosclerosis, as P2Y2 null mice are protected against the disease [81].

Activation of endothelial P2Y2 also has been linked to the mechanism by which oxidized LDL promotes vascular inflammation in atherosclerosis. P2Y2 up-regulates cell adhesion molecules such as VCAM-1 and ICAM-1 to secure monocyte adherence and infiltration into the atherosclerotic lesion [82]. P2Y6 also plays a pro-inflammatory pathogenic role in the disease, with its expression increasing in atherosclerotic lesions [82]. P2Y6 null mice had decreased inflammation associated with lesions, and the macrophages prevent uptake of less cholesterol [82]. These results indicate that P2Y6 antagonists may be beneficial to protect against atherosclerosis.

Additional evidence suggests that GPR120, a GPCR for omega-3 fatty acid, seems to counteract oxidized-LDL/P2Y2-mediated inflammation in atherosclerosis, inhibiting the binding of monocytes to ECs [83]. GPR120 is downregulated by exposure to oxidized-LDL, suggesting a role for GPR120 in mediating oxidized-LDL insult. The activation of GPR120 inhibits ROS and cytokine generation induced by oxidized-LDL by elevating the KLF2 transcription factor’s expression level. KLF2 has been determined to exhibit vascular protective actions by inducing eNOS and thrombomodulin and inhibiting VCAM-1 and E-selectin expression [84]. In addition, a pro-thrombotic state is a part of endothelial dysfunction. Activation of P2Y2 leads to a pro-thrombotic state by up-regulating tissue factor (TF), the initiator of thrombosis [85,86].

#### 2.2.4. Metabolic Syndrome

It is thought that in a hyper-adiposity state associated with metabolic syndrome, a high turnover of adipocytes is observed despite increased fat content. Death of adipocytes promotes sterile inflammation, and this is the connection to CVD. ATP, being released from EC and other cell type, is pro-inflammatory and instigates leukocyte infiltration via P2Y2 receptors. P2Y2-null mice show blunted responses on a high-fat diet, gain less weight, and have better insulin sensitivity and lower cholesterol levels [81]. Adipose tissue is highly vascularized, and some of the P2Y2 action probably spills over to effects on EC P2Y2.

### 2.3. P2Y Receptors and Regulation of Vascular Barrier Function

Increased vascular permeability is an important pathophysiological component of many CVD and lung diseases, including acute lung injury (ALI) and its more severe form, acute respiratory distress syndrome (ARDS), which arise from a wide range of lung injuries such as toxins or inflammatory mediators, resulting in significant morbidity and frequently in death [87,88,89]. In addition, thrombotic events, like stroke, myocardial infarction, and pulmonary embolism, are associated with significant risk factors of lung injury [90,91,92,93]. A major cause of ALI is a dysfunction of the pulmonary vascular endothelial barrier resulting in pulmonary infiltrates, hypoxemia, and pulmonary edema [87]. Recent studies suggest a dual role of P2Y receptor signaling in vascular barrier regulation.

#### 2.3.1. P2Y Receptors and Vascular Permeability

In addition to the regulation of vascular tone, P2Y1,2,4,6 receptors seem to potentiate vascular inflammation and increase vascular leak, decreasing the barrier function of the endothelium [94]. It has been shown that vascular leak is transient when only stimulated by P2Y1,2,4,6-receptor-mediated activation of Gαq-PLC, and much longer-lasting responses occur when ECs are co-stimulated with P2Y2 and LPS or other pro-inflammatory cytokines. Other GPCR ligands that mediate Gq-PLC activation, such as thrombin, histamine, PAF, and bradykinin, also transiently increase vascular permeability. P2Y1-mediated EC permeability involves the activation of CaMKIIδ6, the predominant CaM-activated protein kinase in ECs [95]. Similarly, Gαq-coupled thrombin receptor PAR1 activates CaMKIIδ6 to promote EC permeability. This pathway involves the activation of the RhoA-ROCK, but not ERK1/2; however, at higher concentrations, thrombin activates additional kinases [95]. These data imply that P2Y1-mediated activation of CaMKII is likely responsible for EC barrier dysfunction.

#### 2.3.2. P2Y Receptors and Vascular Barrier Protection

**Figure 1:**
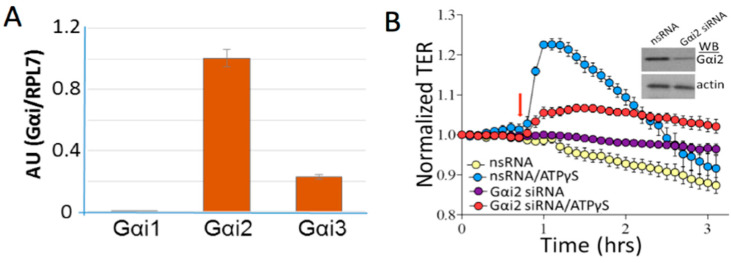
P2Y4 and P2Y12 mediate lung vascular barrier function. (**A**): Quantitative real-time polymerase chain reaction (qPCR) analysis of Gαi in lung microvascular endothelial cells; (**B**): depletion of Gαi2 attenuated ATPγS-induced increase of transendothelial electrical resistance (TER). Human lung microvascular endothelial cells (HLMVECs) were transfected with non-specific siRNA (nsRNA) or Gαi-specific siRNAs (50 nM, 72 h) and were treated with ATPγS (100 µM, arrow). Inset: Western blot analysis shows the efficiency of Gαi depletion.

Although the role adenosine, the ligand for P1 receptors, in the enhancement of endothelial barrier function has been established [96,97,98,99,100], less is known about the effects of ATP. Recent findings in lung microvascular EC presented new evidence on the role of P2Y receptors in vascular barrier protection. It was demonstrated that ATP and its stable analog, ATPγS, can significantly enhance the human lung microvascular EC (HLMVEC) barrier via P2Y4 and P2Y12 receptors coupled to Gq and Gi heterotrimeric proteins [24,101]. Quantitative real-time polymerase chain reaction (qPCR) analysis demonstrated the expression of all three Gαi subtypes in HLMVEC with Gαi_2_ expressed at the highest and Gαi_1_ at the lowest (almost negligible) levels (Figure 1A). Specific depletion of Gαi_2_ significantly attenuated the ATPγS-induced increase in the trans-endothelial electrical resistance (TER), an inverse permeability index (Figure 1B) confirming the involvement of Gi_2_ in the ATPγS-induced barrier enhancement. In addition, it was shown that ATPγS attenuates the gram-negative bacterial toxin LPS-induced lung inflammation and vascular leak in vivo [35]. While the mechanisms involved in ATPγS-induced EC barrier preservation remain ill-defined, some data suggest that they likely include unconventional Gi-mediated PKA activation and specific association of Gi and PKA with AKAP2 (PKA anchoring protein 2) [24]. In this signaling cascade, downstream mechanisms include the activation of myosin light chain (MLC) phosphatase (MLCP), followed by dephosphorylation of MLC, leading to inhibition of contractile responses and enhancing of cell-cell junctions [24,101]. Importantly, depletion of Gαi_2_ reversed ATPγS-induced MLC dephosphorylation, suggesting the involvement of P2Y4/P2Y12/Gi2-mediated signaling in MLCP activity regulation [24].

Nicotinamide adenine dinucleotide (NAD) is a cofactor that is central to metabolism. In addition to its metabolic functions, NAD^+^ emerges as an adenine nucleotide that can be released from neurons and blood vessels spontaneously and by regulated mechanisms [102,103]. In recent years, NAD^+^ has also been recognized as an extracellular signaling molecule involved in cell-to-cell communication [102,104,105]. Recent in vitro studies demonstrated that extracellular β-nicotinamide adenine dinucleotide (β-NAD) protects the pulmonary endothelial cell barrier integrity from injury caused by thrombin and bacterial toxins, LPS, and pneumolysin (PLY) [36]. In addition, β-NAD induced rearrangement of the adherens junction protein VE-cadherin, supporting a tightening of the cell–cell contacts and strengthening of endothelial barrier function. Pharmacological and genetic inhibitory approaches in human pulmonary artery EC revealed the participation of P2Y1 and P2Y11 receptors in β-NAD-induced TER increases. The signaling mechanisms involve the activation of PKA, EPAC1 (the cAMP activated GEF for Rap1), small GTPase Rac1, and MLCP [17,36]. In addition, evidence suggests that P2Y11-mediated activation of EPAC1 opposes the pro-inflammatory action of RhoA-ROCK via Tiam-and Vav-mediated Rac activation that strengthens the endothelial adherens junction leading to the prevention of vascular leak and inflammation [77].

**Figure 2:**
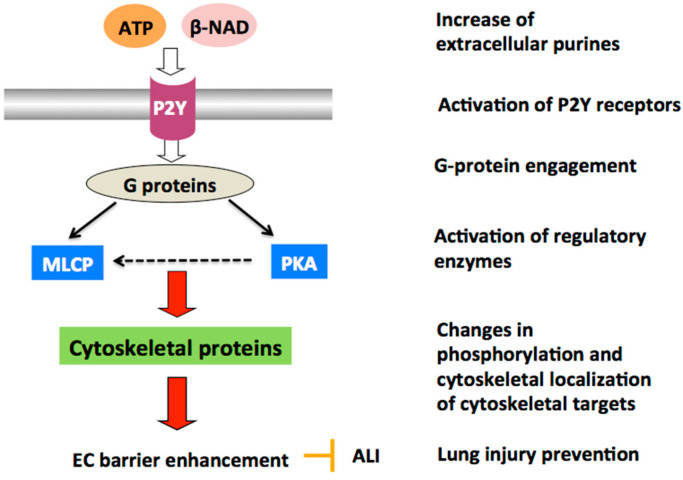
P2Y-mediated mechanisms of lung endothelial barrier protection. P2Y agonists (ATP, ATPγS, or β-NAD) enhance endothelial barrier properties via consequential activation of heterotrimeric G-proteins and enzymes, like myosin light chain phosphatase (MLCP) and PKA, followed by cytoskeletal rearrangement, increased cell-cell contacts, and tightening of endothelial cell (EC) barrier, thus opposing acute lung injury progression.

Consistent with the in vitro studies, β-NAD attenuated the LPS-induced inflammatory cells infiltration and decreased permeability in the lungs of LPS/β-NAD-treated mice compared to mice treated with LPS alone. Histological specimens from the lung showed increased, and prominent interstitial edema with infiltration of neutrophils in the lung parenchyma of mice treaded with LPS alone compared to the LPS/β-NAD-treated lungs [37]. Therefore, similar to ATP and ATPγS, P2Y agonist β-NAD is a potent positive regulator of the pulmonary endothelial barrier integrity both in vitro and in vivo (Figure 2).

### 2.4. P2Y Receptors and Regulation of Vessel Growth

Purinergic regulation of vascular growth and development is a rapidly developing concept in vascular biology, focusing on the role of specific P2YR subtypes. In addition to vasoprotective vasodilation, P2Y receptors are critical to angiogenesis, vasculogenesis, and endothelial differentiation. Several cell and animal studies have provided evidence on the importance of nucleotide signaling for vascular growth. A study on P2Y4 knockout mice demonstrated a role for this receptor in cardiac micro-vessel growth, migration, and PDGF-B secretion in response to UTP. This study also showed that P2Y4 is an essential regulator of endothelial-cardiomyocyte interactions in post-natal heart development [106]. Decreased expression of CD39/ENTPDase, the enzyme responsible for extracellular ATP degradation, was observed in plexiform lesions from PAH patients. It was hypothesized that increased extracellular ATP level in the pulmonary vascular endothelium, as well as within the angiomatoid proliferative lesions might promote excessive endothelial proliferation [107]. Importantly, functional crosstalk was observed between purinergic and growth factor receptors. For example, P2Y1 operates via transactivated vascular endothelial growth factor receptor (VEGFR2) in angiogenic signaling [108]. ATP, ADP, and their synthetic analogs (ATPγS, ADPβS, MeSATP, MeSADP) in combination with platelet extract growth factors synergistically increased the number of functional neovessels in matrigel plugs subcutaneously injected in mice [109].

#### 2.4.1. *Vasa Vasorum* Neovascularization

Proliferative vascular remodeling has a central role in the pathology of many cardiovascular diseases [110,111,112,113,114,115,116]. *Vasa vasorum* (VV) is the microcirculatory network of the bronchial (systemic) circulation and, similar to its role in systemic vessels, contributes to vascular integrity through the supply of oxygen and nutrients to the outer part of the pulmonary artery (PA) wall. Studies on animal models of PH revealed marked adventitial thickening and expansion of the VV network, which are especially prominent components of the pulmonary vascular remodeling process [117,118]. An increasing body of experimental data has demonstrated that the expansion of the VV might also contribute to the progression of certain vascular diseases in systemic circulation including atherosclerosis, restenosis, vasculitis, and ascending aortic aneurism suggesting that neovascularization of the VV might be an important common feature of specific pulmonary and systemic vascular diseases [119,120,121,122,123,124,125]. Expansion of the bronchial vessels in the ischemic lung parenchyma and the PAs has also been demonstrated in patients with chronic thromboembolic disease, which suggests a unique proliferative and invasive capacity VV endothelial cell (VVEC) [126]. At present, the precise cellular mechanisms and endogenous molecular factors contributing to this process of neovascularization in the vessel wall are not entirely understood. Considering that within the PA adventitial compartment, ATP can be released as a result of the combined action of hypoxia, inflammation, mechanical forces, and sympathetic stimulation, elevated extracellular ATP levels may contribute to purinergic regulation of the pulmonary artery VV neovascularization [118].

#### 2.4.2. Angiogenic Purinergic Signaling in VVEC

The findings from a neonatal bovine model of PH demonstrated that ATP is a potent angiogenic factor for PA VVEC [12,127]. Moreover, comparative studies on the pro-angiogenic effects of ATP in various EC subtypes revealed that extracellular ATP is a more potent mitogen for microvascular ECs (VVEC and lung microvascular EC) than for ECs of large vessels (main PA and aorta EC) [127]. It can be speculated that VVEC in the hypoxic PA adventitia has distinct phenotypic characteristics, with a particular reliance on extracellular nucleotides as pro-angiogenic stimuli. The exceptional sensitivity of microvascular ECs to stimulation with ATP and ADP is supported by other studies showing a mitogenic effect of P2Y agonists like ATP, UTP, and MeSATP on brain capillary and corneal endothelial cells [128,129]. Similarly, it was found that ATP and the other P2Y agonists exerted only weak increases in DNA synthesis in bovine aortic EC, supporting the idea that purinergic signaling has little impact on differentiated endothelial cell proliferation [130,131,132]. In addition, PA adventitial VVEC are a potent source of extracellular ATP, which is released via regulated exocytosis and acts as an autocrine/paracrine factor augmenting hypoxia-induced VVEC angiogenesis [12,25,127]. Pharmacological and genetic (siRNA) approaches revealed that the angiogenic effects of extracellular nucleotides in VVEC are mediated through dramatic and prolonged activation of P2Y1 and P2Y13 receptors and PI3K/mTOR and ERK1/2 pathways, as well as the elevation of cytoplasmic and nucleoplasmic Ca^2+^, [25,127,133,134,135,136,137,138]. Recent studies on VVEC also demonstrated a critical role of the P2Y-PI3K-Akt-mTOR axis in the activation of c-Jun, c-Myc, and Foxo3a transcription factors; showed a functional significance of these proteins in VVEC angiogenic responses; and identified target genes involved in tissue remodeling, cell cycle control, cell adhesion, and barrier function [69]. Therefore, data on VVEC and lung microvascular EC provide critical evidence on the regulatory role of extracellular nucleotides in angiogenesis [19,109,127,139]. Considering that the responses to extracellular ATP might be particularly important in the hypoxic and inflamed adventitial microenvironment, targeting P2Y receptors may have a potential translational significance in attenuating pathological vascular remodeling associated with many CVDs.

#### 2.4.3. P2Y Receptor Subtypes and VV Neovascularization

**Figure 3:**
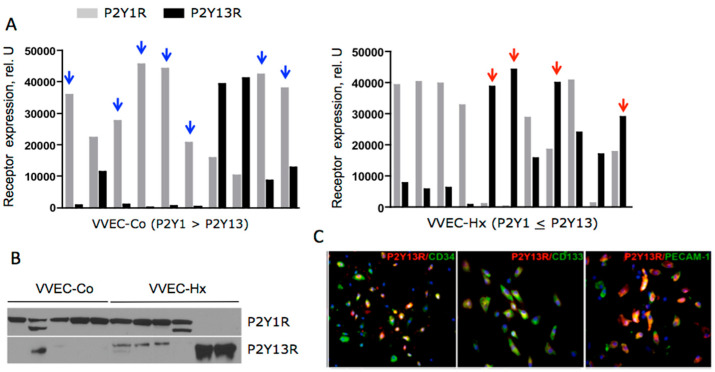
P2Y1 and P2Y13 receptor expression in pulmonary artery vasa vasorum endothelial cells (VVECs). Cells were isolated from the pulmonary arteries of control and chronically hypoxic hypertensive calves. (**A**,**B**): WB analysis of P2Y1R and P2Y13R expression in VVEC-Co and VVEC-Hx. Blue arrows indicate increased expression of P2Y1R compared to P2Y13R in some VVEC-Co populations; red arrows indicate increased expression of P2Y13 compared to P2Y1R in some VVEC-Hx populations; (**C**): Immunofluorescent analysis of P2Y13 (red) co-expression with CD31/PECAM, CD34, and CD133 (green) in VVEC-Hx.

**Figure 4:**
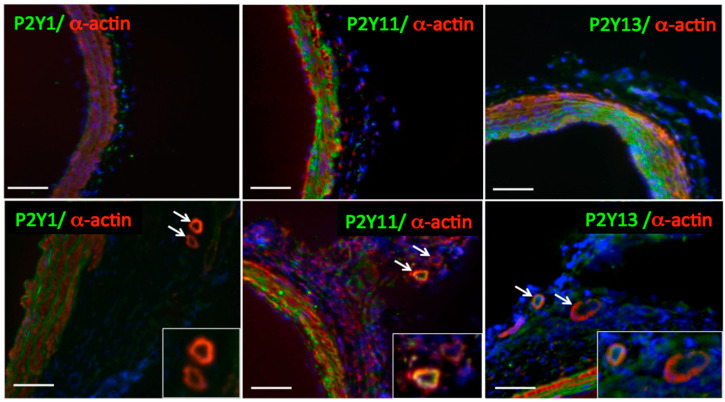
P2Y1, P2Y11, and P2Y13 purinergic receptors are expressed in angiogenic pulmonary artery *vasa vasorum***.** Immunofluorescent analysis of frozen acetone/methanol-fixed tissue sections of control and chronically hypoxic rats (4 weeks, (barometric pressure) P_B_ 430 mmHg) revealed pulmonary artery adventitial *vasa vasorum* expansion (white arrows) and the expression of P2Y1, P2Y11, and P2Y13 receptors (green), and α-actin (red) in chronically hypoxic, but not control animals; scale bar = 100 μm.

As indicated above, pulmonary artery VV angiogenic expansion is a characteristic feature of hypoxia-induced pulmonary vascular remodeling in PH. The expression of P2Y13 has been previously shown in spleen, brain, and circulating CD34+ progenitor cells, but not in vascular endothelium [9,140]. The involvement of P2Y13 in VVEC proliferation is intriguing. Studies on VVEC isolated from PA adventitia demonstrated the expression of P2Y1 (known as an endothelial-specific purinergic receptor) in most VVEC populations isolated from the PA of control (VVEC-Co) and hypoxic (VVEC-Hx) animals, in contrast, the expression of P2Y13 (known as a progenitor cell receptor) was observed to a more considerable extent in VVEC-Hx (Figure 3A,B). Furthermore, the heterogeneous pattern of P2Y1 and P2Y13 expression together with co-expression of P2Y13 with progenitor cell markers CD31/PECAM-1, CD133, and CD34 observed in VVEC-Hx (Figure 3C) suggests that hypoxia-induced VV angiogenic expansion may involve the emergence of P2Y13 expressing highly proliferative progenitor-like cell populations residing in the VV [117]. The expression of P2Y13, well as P2Y1 and P2Y11, was also observed in the VV of the PA adventitia chronically hypoxic Sprague Dawley rats, supporting a potential involvement of these receptors in endothelial differentiation and angiogenesis (Figure 4). Evaluation of the P2Y13 involvement in endothelial angiogenic responses would validate these receptors as novel pharmacological targets for VV neovascularization and pathologic vascular remodeling in PH and other cardiovascular diseases.

### 2.5. P2Y Receptors and Infantile Hemangioma Development

**Figure 5:**
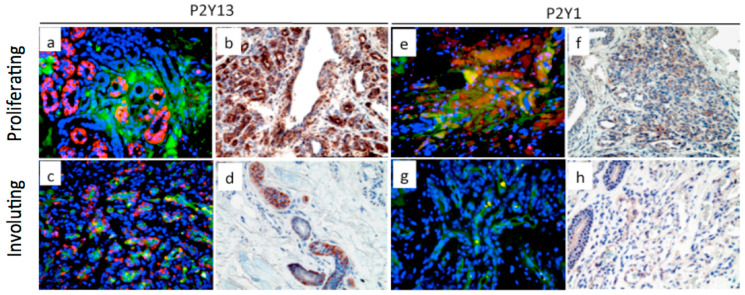
P2Y13 and P2Y1 receptor expression in proliferating and involuting infantile hemangiomas. (**a**,**c**,**e**,**g**): Double immunofluorescent analysis for P2Y13 and P2Y1 receptors (Alexa 594, red) and PECAM/CD31 (Alexa 488, green); nuclei were counterstained with DAPI (blue; Carl Zeiss, x40 magnification; (**b**,**d**,**f**,**h**): Immunohistochemical analysis for P2Y13 and P2Y1 receptors (AEC substrate, Olympus, X40 magnification). Tissue sections were kindly provided by Dr. Sule Cataltepe (Children’s Hospital, Boston, MA, USA).

Infantile hemangiomas are vascular tumors characterized by excessive proliferative/dysregulated endothelial proliferation and spontaneous involution. However, the mechanisms underlying their unique “lifecycle” remain largely unknown. Previous studies on hemangiomas have been restricted by elucidating canonical angiogenic pathways that include vascular endothelial growth factor (VEGF) and its receptors VEGFR1 and VEGFR2, IGF-I, Tie-1, Tie-2, bFGF, and angiopoietin [141]. Considering that P2Y-mediated signals are essential for the coordinated cell proliferation, migration, and differentiation in the developing organs and tissues [9,18,142], and the alteration of P2 receptor subtype expression can be implicated in pathologic cellular responses in the vasculature [9,18,143,144,145], it can be expected that hemangioma EC growth and/or regression may be regulated by extracellular nucleotides. Immunohistochemical analysis demonstrated the expression of P2Y13 in both proliferating and involuting infantile hemangiomas (Figure 5). Remarkably, expression of P2Y13 in proliferating hemangiomas was dramatically higher compared to those observed in involuting hemangiomas, suggesting that P2Y13 may be functionally involved in hemangioma expansion. Similar to P2Y13, P2Y1 expression was found to be higher in proliferating hemangiomas. It was also shown that involuting hemangiomas exhibit an overlapping pattern of the expression of endothelial marker CD31/PECAM and P2Y1, indicating that decreased hemangioma vascularity correlates with P2Y1 expression level. These data suggest that the expression of P2Y1 might be more attributable to a more differentiated endothelial phenotype, whereas the expression of P2Y13 is related to less-differentiated progenitor-like phenotype.

## 3. Conclusions and Perspectives

**Figure 6:**
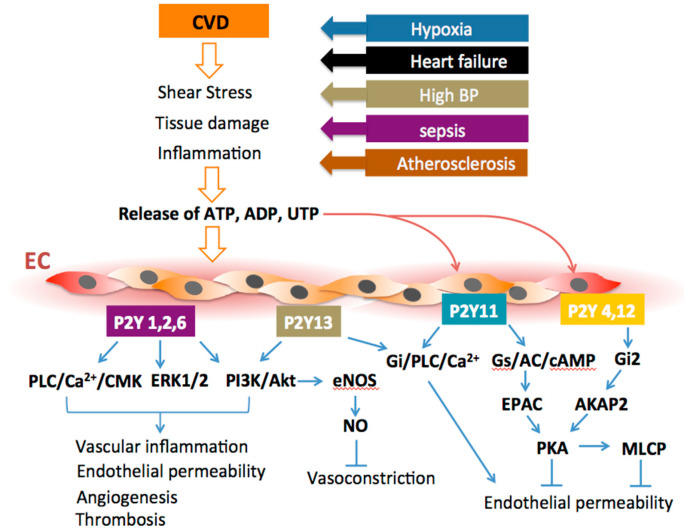
Overview of the molecular mechanisms and endpoints on the involvement of endothelial P2Y receptors in cardiovascular disease (CVD). Pathological responses to CVD include the release of extracellular purines at the sites of injury, following by activation of P2Y receptors and specific P2YR-mediated intracellular pathways leading to either injury progression, resolution, or both in tissue- and agonist-specific manner.

Endothelial purinergic signaling in CVD remains the area of intensive investigation. With the growing interest in pathological vascular remodeling, inflammation, angiogenesis, acute lung injury, stem cell differentiation, and vascular tumors, the regulatory circuits of extracellular nucleotides that operate via the activation of purinergic receptors may bring a new dimension to a better understanding of CVD mechanisms (Figure 6). Research on purinergic receptor signaling in inflammatory, neuronal, and cancer cells significantly advanced the understanding of the physiological and pathological purinergic signaling mechanisms in many diseases. In contrast, fewer studies have been focused on purinergic signaling in vascular bed-and organ-specific endothelium, though this research direction may uncover previously overlooked involvement of P2Y receptors in endothelial function. Studies on P2Y1, P2Y2, P2Y4, and P2Y6, as well as P2Y13 and P2Y13 receptor knock-out mice, revealed the biochemical and functional diversity of these receptors in various organs. In perspective, a combination of the genetic and advanced pharmacological approaches could recognize vascular bed-specific expression and function of P2Y receptor subtypes and distinguish their contribution to endothelial physiology in CVD. It should also be acknowledged that advances in medicinal chemistry and high-throughput screening of novel P2Y agonists and antagonists will identify the new therapeutic application for targeting P2Y receptors. An existing challenge now relates to identifying purinergic antagonists for therapeutic use, as the most purinoceptors have many polymorphic variations. There is also a need for developing orally bioavailable and stable in-vivo purinergic compounds. A better understanding of R2Y receptor covalent modifications, homo-and hetero-dimerization with purinergic and, possibly, other G-protein-coupled receptors will promise exciting and vital observations in the future. It would not be surprising if purinergic signaling mechanisms, like the one of the most conservative, fundamentally important, and ubiquitous, would be re-discovered as clinically relevant regulatory mechanisms in vascular endothelium.

## Abbreviations

AC	Adenylate cyclase
ADP	Adenosine triphosphate
ADPβS	Adenosine-5′-0-(2-thiodiphosphate)
AKAP2	PKA anchoring protein 2
Akt	Serine/threonine protein kinase
ALI	acute lung injury
ApoE	Apolipoprotein A
ARDS	Acute respiratory distress syndrome
ATPγS	Adenosine 5′-(3-thiotriphosphate)
AT1R	Angiotensin II type I receptor
CAM	Cell adhesion molecule
CaMKII	Calmodulin kinase
cAMP	Cyclic adenosine monophosphate
CD39/ENTPDase	Ectonucleoside triphosphate diphosphohydrolase
CVDs	Cardiovascular diseases
Cx 37, 40	Connexin 37, 40
DAMP	damage-associated molecular pattern
EC	Endothelial cells
ECM	Extracellular Matrix
ED	Endothelial dysfunction
EDH	Endothelium-dependent hyperpolarization
eNOS	Endothelial nitric oxide synthase
EPAC	Exchange Protein Activated by cAMP
ERK1/2	Extracellular signal regulated kinases 1/2
E-selectin	CD62 antigen-like family member E
GEF	Guanine nucleotide exchange factor
GPCR	G-protein coupled receptor
GPR120	G-protein coupled receptor 120
Gs, Gs	G protein subtypes
ICAM-1	Intercellular Adhesion Molecule 1
IGF-I	Insulin-like growth factor 1
IL-1, -6	Interleukin-1, -6
JNK	c-Jun N-terminal kinase
KCa3.1	The calcium-activated potassium channel KCa3.1
KLF	The Krüppel-like family of transcription factors
LDL	Low-density lipoproteins
LMVEC	Lung microvascular endothelial cells
LPS	Lipopolysaccharide
MeSADP	2-(Methylthio)adenosine 5’-diphosphate
MeSATP	2-(Methylthio)adenosine 5′-triphosphate
MLC	Myosin light chains
MLCP	MLC phosphatase
MMPs	Matrix metallopeptidases
mTOR	The mammalian target of rapamycin
β-NAD	β-Nicotinamide adenine dinucleotide
NFkB	nuclear factor kappa-light-chain-enhancer of activated B cells
NO	Nitric oxide
NOX2	NADPH oxidase catalytic component
PAF	Platelet-activating factor
P_B_	Barometric pressure
PIEZO	Mechanically activated cation channels
PGI_2_	Prostacyclin
PKA	Protein kinase A
PKC	Protein kinase C
PKG	Protein kinase G
PLC	Phospholipase C
PLY	Pneumolysin
PI3K	Phosphatidyl inositol 3 kinase
PAH	Pulmonary arterial hypertension
p38 MAPK	p38 Mitogen-activated protein kinases
Rac 1	A small GTPase protein
Rap 1	A small GTPases protein
ROS	Reactive oxygen species
ROCK	Rho protein kinase
TER	Transendothelial electrical resistance
Tiam 1	T-Lymphoma invasion and metastasis-inducing protein 1
TNFαR	Tumor necrosis factor alpha receptor
TXA2	Thromboxane A2
TLR	Toll-like receptor
TRPV4	Transient receptor potential cation channel subfamily V member 4
T1D, T2D	Type 1,2 diabetes
UTP	Uridine triphosphate
Vav	Guanine nucleotide exchange factor
VCAM-1	Vascular cell adhesion protein 1
VEGFR2	Vascular endothelial growth factor receptor-2
VSMC	Vascular smooth muscle cell
VVEC	Vasa vasorum endothelial cells
